# Effects of Dietary *Saccharomyces cerevisiae* YFI-SC2 on the Growth Performance, Intestinal Morphology, Immune Parameters, Intestinal Microbiota, and Disease Resistance of Crayfish *(Procambarus clarkia)*

**DOI:** 10.3390/ani11071963

**Published:** 2021-06-30

**Authors:** Yan Xu, Yiqun Li, Mingyang Xue, Tao Yang, Xiaowen Luo, Yuding Fan, Yan Meng, Wenzhi Liu, Ge Lin, Bo Li, Lingbing Zeng, Yong Zhou

**Affiliations:** 1 Yangtze River Fisheries Research Institute, Chinese Academy of Fishery Sciences, Wuhan 430223, China; xy13033732426@163.com (Y.X.); liyq@yfi.ac.cn (Y.L.); xmy@yfi.ac.cn (M.X.); m18790069727@163.com (X.L.); fanyd@yfi.ac.cn (Y.F.); mengy@yfi.ac.cn (Y.M.); liuwenzhialisa@yfi.ac.cn (W.L.); linge0310@163.com (G.L.); 2 Animal Health Research Institute, Tongwei Co., Ltd., Chengdu 610041, China; yangt@tongwei.com; 3 Wuhan Academy of Agricultural Science, Wuhan 430207, China; libowuhan003@163.com

**Keywords:** crayfish, growth, immune, intestinal microbiota, intestinal morphology, *Saccharomyces cerevisiae*

## Abstract

**Simple Summary:**

Diseases of crayfish (*Procambarus clarkia*) are closely related to intestinal health. Therefore, it is important for crayfish aquaculture to keep intestinal health in an optimum condition. As a beneficial fungus, *Saccharomyces cerevisiae* can effectively compete to inhibit the reproduction of pathogenic bacteria, regulate the intestinal microecosystem, and promote animal growth and disease resistance. This study aimed to assess the effects of *S. cerevisiae* YFI-SC2 on the growth, immunity, and intestinal health of crayfish. The results demonstrated that the addition of *S. cerevisiae* to the feed could improve the growth performance, the immune response, the intestinal morphology, the structure of intestinal microbiota, and the resistance to pathogens of crayfish. Therefore, *S. cerevisiae* can be used as a potential probiotic in crayfish farming.

**Abstract:**

The present study aimed to evaluate the effect of the dietary supplementation of *Saccharomyces cerevisiae* YFI-SC2 on the growth performance, intestinal morphology, immune parameters, intestinal microbiota, and disease resistance of crayfish (*Procambarus clarkia*). Crayfish were randomly assigned to six different boxes and two different groups in triplicate. The control group received a basal diet and the treatment group received a diet containing *S. cerevisiae* at 10^7^ CFU/g. After feeding for 28 days, crayfish of the treatment group exhibited a significantly better weight gain ratio (WGR) and a specific growth rate (SGR) (*p* < 0.05) than crayfish of the control group. Compared to the treatment group, the control group intestines showed an oedema connective tissue layer and a weak muscle layer. For immune-related genes, Crustin2 expression was similar between the groups, whereas Lysozyme and prophenoloxidase from treatment group expression levels were upregulated significantly (*p* < 0.05) after 14 and 28 days of feeding. Prophenoloxidase showed the highest expression, with 10.5- and 8.2-fold higher expression than in the control group at 14 and 28 days, respectively. The intestinal microbiota community structure was markedly different between the two groups. After 14 and 28 days of feeding, the relative abundance of *Cetobacterium* and *Lactobacillus* increased, whereas *Citrobacter* and *Bacteroides* decreased in the treatment group compared with that of the control group. The challenge test showed that crayfish of the treatment group had a significantly enhanced resistance against *Citrobacter freundii* (*p* < 0.05). Our results suggest that a *S. cerevisiae*-containing diet positively influenced the health status, immune parameters, intestinal microbiota composition, and disease resistance of crayfish.

## 1. Introduction

Crayfish belong to the subphylum Crustacea, the order Decapoda, and the family Cambaridae [1]. Crayfish is native to eastern North America. It was first introduced to Japan in 1918, and then entered China in the 1930s [2]. Its bright color and delicious taste mean that crayfish are loved by consumers, and they have recently become a commercially important freshwater species in China [3]. In 2018, world production of crayfish was 1.71 million tons [4], and it was 1.64 million tons in China [5]. Comparing with that in 2018, China’s total production of crayfish reached 2.09 million tons, with an increase of 27.52% in 2019 [5]. However, with the development of the industry, the reports on the occurrence of crayfish diseases were also gradually increased. *C. freundii*, as a typical conditional pathogen, has strong pathogenicity and lethality toward crayfish. This may result in economic losses to the crayfish aquaculture industry [6]. Many crayfish diseases are closely related to intestinal health, especially the structure of the intestinal microbiota [7]. The intestinal microbiota’s structural composition, diversity, and stability are important factors affecting host health [8]. Meanwhile, as invertebrates, the innate immune system of crayfish plays an important role in resistance to disease [9]. Toll-like receptors and prophenoloxidase are important parts of the innate immune signal recognition and transduction system [10]. Crustin2 and lysozyme are classes of antibacterial peptides with broad-spectrum antibacterial activity that have important antibacterial and antiviral effects [11,12]. Therefore, maintaining the dynamic balance of the intestinal microbiota and promoting immune gene expression are important for the healthy development of the crayfish industry.

Probiotics have been proven to be beneficial in improving aquatic animal health and immunity via modulation of the intestinal microbiota [13]. They can also improve feed digestibility and the absorption rate, promote healthy intestinal morphology, and enhance disease resistance [14]. *S. cerevisiae* is a beneficial fungus that can effectively compete with and inhibit the reproduction of pathogenic bacteria in the process of proliferation. In addition, *S. cerevisiae* can produce cell wall polysaccharides, active bacteria, minerals, vitamins, and growth-promoting factors during the fermentation process, which favor the improvement of nutrient digestion, promote the development of the intestinal mucosa, and maintain the balance of the intestinal microbiota in animals [15,16,17]. Studies have shown that *S. cerevisiae* can promote the growth, immunity, and disease resistance, and can improve the intestinal microbiota structure, of gibel carp (*Carassius auratus gibelio*), common carp (*Cyprinus carpio*), and *Arapaima gigas* [17,18,19]. Thus, *S. cerevisiae* provides an important reference for the development of probiotics in aquatic products. A number of studies verified the beneficial effects of *S. cerevisiae* on white shrimp (*Litopenaeus vannamei*) [20,21]; however, data regarding the effects of probiotic bacteria or *S. cerevisiae* on the growth, immunity, and intestinal health of crayfish are limited.

In the present study, we aimed to add *S. cerevisiae* YFI-SC2 to crayfish feed and study the effect of *S. cerevisiae* YFI-SC2 on growth performance, intestinal morphology, the immune response, and the intestinal microbial community composition, to provide a useful reference for the development of the crayfish industry.

## 2. Materials and Methods

### 2.1. Experimental Animals

The experimental crayfish (13.71 ± 0.58 g) were obtained from a local farm in Qianjiang, Hubei, China. Crayfish were temporary cultured in breeding boxes (75 cm × 50 cm × 25 cm) for 7 days before the feeding experiments. Each breeding box was filled with 25 L of fresh water (pH 7.8 ± 0.2, temperature 23 ± 1 °C, dissolved oxygen 6.5 ± 0.5 mg/L) and contained two evasion devices (25 cm × 20 cm × 12 cm, unsealed at one end). Approximately 50% of the water was exchanged from each box on a daily basis along with the siphoning of fecal matter from the bottom. The experimental water was tap water that was aerated for more than 48 h. The crayfish were fed commercial pellets supplied by Tongwei Co., Ltd., Chengdu, China (≥28% protein, ≥3% fat, ≤12% water, and ≤18% ash) twice daily (9:00 and 17:00). The crayfish were fed with 1.5% of their total body weight. All experimental procedures were conducted according to guidelines of the appropriate Animal Experimental Ethical Inspection of Laboratory Animal Centre, Yangtze River Fisheries Research Institute, Chinese Academy of Fishery Sciences (ID Number: YFI2021-zhouyong-01).

### 2.2. Yeast Strains and Growth Conditions

*S. cerevisiae* YFI-SC2 was isolated from the intestinal tract of crayfish and stored at the China Center for Type Culture Collection (CCTCC), Wuhan University, whose preservation number was CCTCC M2021311. *S. cerevisiae* was cultured overnight in yeast peptone dextrose adenine (YPDA; Qingdao biological technology Co., Ltd., Qingdao, China) at 30 °C and resuspended in sterilized phosphate-buffered saline (PBS). The colony forming units (CFU) per milliliter of *S. cerevisiae* culture was determined by the plate dilution counting method. The *S. cerevisiae* resuspended cells were added to the commercial basal feed at 10^7^ CFU/g of feed [21]. The feed was vacuum-dried at 30 °C overnight and stored at 4 °C. The feed was prepared in this way every 3 days.

### 2.3. Feeding Frequency

After 7 days of acclimation, the experimental crayfish were divided into 6 boxes each containing 80 crayfish. The boxes were randomly divided into the treatment group (TG) and the control group (CG) (three boxes each). The treatment group was fed *S. cerevisiae* feed, and the control group was fed the basal commercial feed for 28 days. In order to clearly explore the probiotic effect of *S. cerevisiae* on crayfish, sampling analysis was conducted on the 14th and 28th days of culturation.

### 2.4. Growth for Performance and Sample Collection

#### 2.4.1. Growth Performance

Weights were measured at the beginning and at the 14th and 28th days of the experiment. The growth parameter indices, the weight gain ratio (WGR), and the specific growth rate (SGR) were used to measure the growth performance of the crayfish. These indicators, and the survival rate, were calculated according to the formulae:

WGR (%) = 100 × (Wf − Wi)/Wi

SGR (% /day) = 100 × (ln Wf − ln Wi)/T

Survival rate (%) = 100 × Ni/Nf

where, the Wf is the weight of the crayfish at sampling; Wi is the initial weight of the crayfish; T is the feeding day when the sample was collected; Ni is the initial number of the crayfish; and Nf is the number of the crayfish at sampling.

#### 2.4.2. Sample Collection

On the 14th and 28th day of the experiment, 8 crayfish were randomly collected per box and aseptically sacrificed in an ice-bath. The anterior intestines were fixed in neutral 4% paraformaldehyde to make tissue sections. The whole intestinal tissue was frozen rapidly in liquid nitrogen and stored at −80 °C for Illumina sequencing (Illumina, San Diego, CA, USA). Hepatopancreases were placed in an RNase-free centrifuge tube with 200 μL TRIzol and stored at −80 °C for quantitative real-time reverse transcription PCR (qRT-PCR).

### 2.5. Histopathology Analysis

The anterior intestine tissues were fixed in 4% paraformaldehyde for 24 h, dehydrated in a sequential series of alcohol (50–95%), and embedded in paraffin. Tissue sections (5 μm thick) were stained with hematoxylin and eosin (H&E). The intestinal structure was analyzed and photographed under a microscope (Olympus BX53 microscope, Tokyo, Japan).

### 2.6. Real-Time PCR Analysis of Immune-Related Genes

Total RNA was isolated using the TRIzol reagent (Invitrogen, Carlsbad, CA, USA) and the residual trace DNA was removed using DNase I (Takara, Dalian, China). Complementary DNA (cDNA) was synthesized using a SuperMix kit (TransGen Biotech, Beijing, China). The cDNA was used as the template for the quantitative real-time PCR (qPCR) step, the reaction mixture for which was comprised of 2 µL of diluted cDNA sample, 10 µL of Power 2 × TB Green Fast PCR Mix (Takara), 0.8 µL of each primer (Table 1) (10 mol/L), and 6.4 µL of H_2_O. The qPCR cycle profile included 1 cycle at 95 °C for 5 min, followed by 40 cycles of 95 °C for 20 s, 55 °C for 20 s, and 72 °C for 20 s. The primers for qPCR are shown in Table 1. Relative gene expression was assessed using the 2^−ΔΔCt^ method [22]. The 18S gene sequence was found on NCBI (MT829236.1), and the primer was designed by primer 5, which was used as the internal control gene for cDNA normalization. All the experiments were repeated three times.

### 2.7. Genomic DNA Extraction and 16S rRNA Gene Sequencing

Bacterial genomic DNA was extracted using a Bacterial DNA Kit (Omega Biotek, Winooski, VT, USA) following the manufacturer’s instructions. PCR amplification of the bacterial V3-V4 region of the 16S rRNA gene was performed in a 50 μL reaction. The reaction contained 25 μL of Hot Start Taq 2 × Master Mix (New England BioLabs Inc., Ipswich, MA, USA), 2 μL of template DNA, 2 μL of each primer (338F and 806R [26], Table 1), and deionized water. The reaction conditions comprised an initial denaturation at 95 °C for 30 s, 30 cycles of 95 °C for 10 s, 55 °C for 30 s, and 72 °C for 30 s, and a final extension step at 72 °C for 5 min. After quality checking using agarose gel electrophoresis, the samples were sequenced on the Illumina MiSeq PE300 high-throughput sequencing platform. All sequencing reads were quality-filtered and assembled using the Mothur software package [27]. The final effective data were obtained using the UCHIME v4.2 software. Reads were clustered into operational taxonomical units (OTUs) at 97% identity [28]. The abundances of the corresponding OTUs in each group were calculated at the phylum and genus levels. Chao 1 (bacterial richness index) and Shannon (bacterial diversity index) alpha diversity indexes were analyzed using Mothur (version v.1.30) [29]. Principle coordinate analysis (PCoA) was used to assess the species differences between samples.

### 2.8. Challenge Test

The pathogenic *C. freundii*, isolated from crayfish intestines and preserved in our laboratory, was used in the challenge test. *C. freundii* was cultured 24 h in brain heart infusion plates (BHI, Qingdao biological technology co., Ltd. Qingdao, China) at 37 °C and resuspended in sterilized phosphate-buffered saline (PBS). After 14 days of the feeding trial, 30 crayfish from each breeding box were immersed in a solution containing *C. freundii* (2.5 × 10^7^ CFU/mL) for 2 h and then transferred to the breeding box. Crayfish mortality was recorded every 24 h for 10 days. After 28 days of the feeding trial, the challenge was carried out again using the same method. The presence of *C. freundii* as the only etiological agent was confirmed by swabbing the intestinal tissues onto BHI plates and subsequent bacteria identification.

### 2.9. Statistical and Correlation Analyses

Statistical analysis was performed using SPSS (version 17.0; IBM Corp., Armonk, NY, USA). One-way analysis of variance (ANOVA) was used to analyze the differences between different treatments, and the LSD test was used to compare means. All data are presented as the mean ± SD. Differences were considered statistically significant at *p* < 0.05.

## 3. Results

### 3.1. Growth Performance

After 14 days and 28 days of feeding, the survival rates of the two groups of crayfish were above 90% (Table 2), and there was no significant difference between the groups. Compared with that in the control group, growth tended to be improved in the treatment group after 14 days of feeding. However, there was no significant difference observed in the WGR and SGR (*p* > 0.05). After 28 days of feeding, the WGR and SGR in the treatment group were significantly higher than those achieved in the control group (*p* < 0.05).

### 3.2. Intestinal Morphology Changes

**Figure 1:**
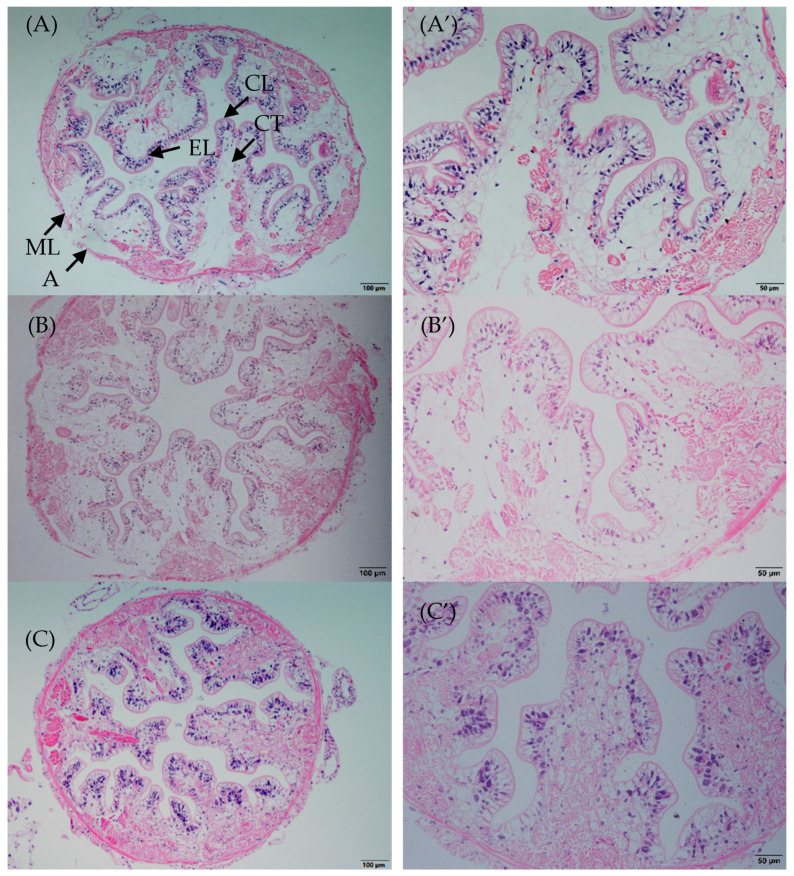

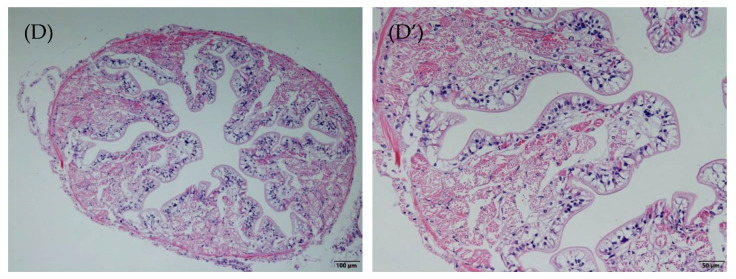
Intestinal morphology analysis of crayfish fed with different experimental diets for 14 days and 28 days. (**A**) control group after 14 days of feeding ((**A**): 100×, (**A’**): 50×); (**B**) control group after 28 days of feeding ((**B**): 100×, (**B’**): 50×); (**C**) treatment group (*S. cerevisiae* at 10^7^ CFU/g diet) after 14 days of feeding ((**C**): 100×, (**C’**): 50×); (**D**) treatment group (*S. cerevisiae* at 10^7^ CFU/g diet) after 28 days of feeding ((**D**): 100×, (**D’**): 50×). CL: chitinous layer; EL: epithelial layer; CT: connective tissue layer; ML: muscle layer; A: adventitia.

After 14 days and 28 days of feeding, the crayfish intestines of treatment group exhibited an orderly and tight chitin layer, epithelial layer, connective tissue layer, muscle layer, and adventitia (Figure 1C,D). By contrast, the intestines of crayfish from the control group showed an oedema connective tissue layer and weak muscle layer (Figure 1A,B)

### 3.3. Expression Levels of Immunity-Related Genes in the Hepatopancreas

**Figure 2:**
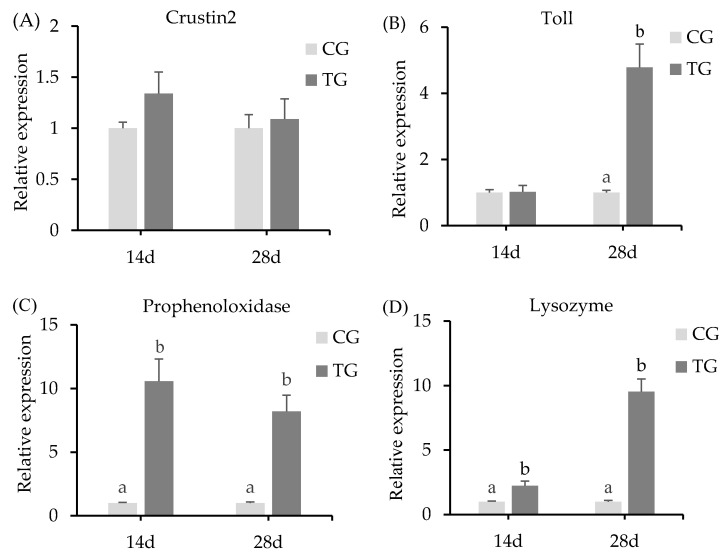
Relative mRNA expression of immunity-related genes in crayfish hepatopancreas tissue after the feeding trial. (**A**): Crustin2 gene expression; (**B**): toll gene expression; (**C**): prophenoloxidase gene expression; (**D**): lysozyme gene expression. Each value represents mean ± SD (*n* = 3), and bars with different letters indicate significant differences by the LSD test for each time point (*p* < 0.05). CG, control group; TG, treatment group (*S. cerevisiae* at 10^7^ CFU/g diet).

The expression levels of immunity-related genes from the crayfish hepatopancreas tissue were detected after treatment with the two different diets, including the genes encoding Crustin2, lysozyme, prophenoloxidase, and the toll-like receptor (Figure 2). Compared with that in the control group, the relative expression of Crustin2 in the treatment group showed no significant difference. However, Lysozyme and prophenoloxidase gene expression levels were upregulated significantly (*p* < 0.05) after 14 days and 28 days of feeding. Prophenoloxidase showed the highest expression, with 10.5- and 8.2-fold higher expression in the treatment group than in the control group at 14 days and 28 days, respectively. There was no significant change in the expression of the toll-like receptor gene after 14 days of feeding. However, it was upregulated significantly (*p* < 0.05) after 28 days of feeding.

### 3.4. Richness and Diversity of Intestinal Microbiota

The 16S rRNA gene amplicons of microbiota were sequenced for the intestines of experimental crayfish. NGS raw data were deposited on NCBI (ID number: RJNA740135). The average number of OTUs obtained from the different samples ranged from 171 to 241, and the coverage rate of OTUs was above 99.9% (Table 3). The results showed that the Chao 1 indexes of TG14d and TG28d were significantly higher than those of CG14d and CG28d (*p* < 0.05). The OTUs and the Shannon index of TG14d were significantly higher than those of CG14d and CG28d (*p* < 0.05).

### 3.5. Phylum and Genus Performance

**Figure 3:**
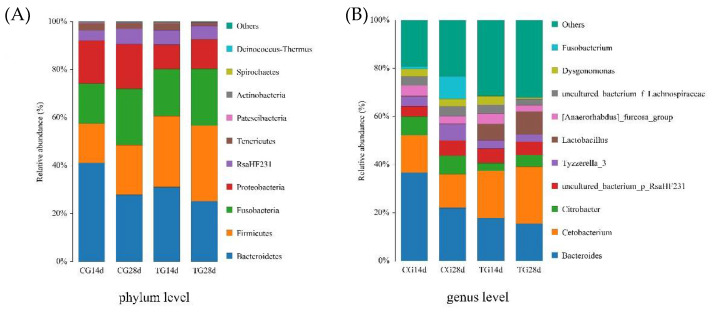
Relative abundance of the intestinal microbiota of crayfish at the phylum (**A**) and genus (**B**) levels. CG14d, control group after 14 days of feeding; CG28d, control group after 28 days of feeding; TG14d, treatment group (*S. cerevisiae* at 10^7^ CFU/g diet) after 14 days of feeding; TG28d, treatment group (*S. cerevisiae* at 10^7^ CFU/g diet) after 28 days of feeding.

To further explore the composition of the microbiota community richness in each group, the abundance of the intestinal microbiota was calculated in each group at the phylum and genus levels for the top ten bacteria. At the phylum level, *Bacteroidetes, Firmicutes, Fusobacteria, and Proteobacteria* were the primary intestinal microbiota in all groups (Figure 3). Compared with that of the control group, the relative abundance of Firmicutes increased after 14 days and 28 days of feeding, whereas the relative abundance of *Bacteroidetes* and *Proteobacteria* decreased in the treatment group. At the genus level, the primary intestinal microbiota in all groups were *Bacteroides*, *Cetobacterium*, *Citrobacter*, and *Lactobacillus* (Figure 3). The relative abundance of *Cetobacterium* and *Lactobacillus* increased in the treatment group, whereas *Bacteroides* and *Citrobacter* decreased, after 14 days and 28 days of feeding compared with that in the control group.

### 3.6. Principle Coordinate Analysis (PCoA)

**Figure 4:**
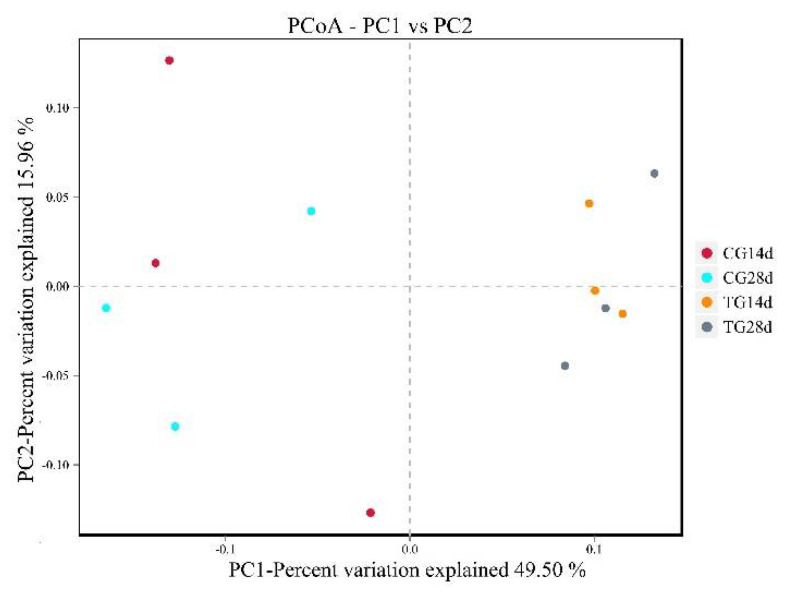
Principal coordinate analysis (PCoA) based on unweighted Unifrac distances. CG14d, control group after 14 days of feeding; CG28d, control group after 28 days of feeding; TG14d, treatment group (*S. cerevisiae* at 10^7^ CFU/g diet) after 14 days of feeding; TG28d, treatment group (*S. cerevisiae* at 10^7^ CFU/g diet) after 28 days of feeding.

The relationships between the microbiota communities from the different groups were evaluated using principal coordinate analysis with the unweighted UniFrac method. After 14 days and 28 days of feeding, the community structure of the intestinal microbiota from the treatment group was distant to that of the control group (Figure 4). In addition, compared with that of the control group, the intestinal microbial community structure of the treatment group was clustered closer after 14 days and 28 days of feeding.

### 3.7. Challenge Test

**Figure 5:**
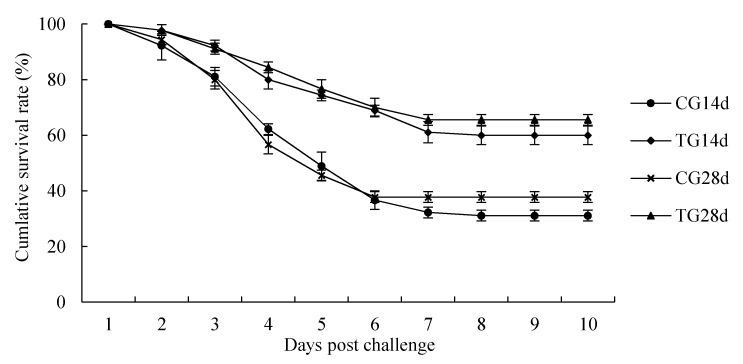
Cumulative survival following a 10-day *C. freundii* challenge in crayfish. Each value represents the mean ± SD (*n* = 3). Different letters were significantly (*p* < 0.05) different by LSD test. CG14d, control group after 14 days of feeding; CG28d, control group after 28 days of feeding; TG14d, treatment group (*S. cerevisiae* at 10^7^ CFU/g diet) after 14 days of feeding; TG28d, treatment group (*S. cerevisiae* at 10^7^ CFU/g diet) after 28 days of feeding.

The cumulative survival rates of crayfish challenged with *C. freundii* for 10-days are shown in Figure 5. During the challenge test, the first mortalities occurred on the second day. At the end of the 10-day challenge test, the cumulative survival rates were 31.11%, 37.78%, 60%, and 65.56% in the CG14d, CG28d, TG14d, and TG28d groups, respectively. Crayfish from the treatment group showed a numerically higher survival rate compared with that in the control group after 14 days and 28 days of feeding.

## 4. Discussion

### 4.1. Growth Performance

Dietary supplementation of probiotics in aquaculture offers an ecofriendly prophylactic measure to improve fish growth performance and health [30]. Probiotics might affect the host beneficially by improving immunity and the intestinal microbial balance [31]. *S. cerevisiae* is an important source of products with probiotic activity [32]. It is rich in nutrients such as protein, functional polysaccharides, amino acids (glutamic acid), and vitamins (Vitamin B) [18]. In particular, the *β*-glucan and mannose oligosaccharides (MOS) in the yeast cell wall could improve the growth performance of giant freshwater prawn (*Macrobrachium rosenbergii*) [33], red sea bream (*Pagrus major*) [34], Chinese mitten crab (*Eriocheir sinensis*) [35], and other aquatic animals [36,37]. In the present study, *S. cerevisiae* at 10^7^ CFU/g in feed showed a growth-promoting effect on crayfish after 28 d, which was similar to the above results.

### 4.2. Intestinal Histomorphometric

In aquatic animals, the intestine is the main organ for digestion and absorption [37]. The normal intestinal epithelial structure also plays an important role in the intestinal barrier function and the mucosal immune response [38,39]. *S. cerevisiae* feed additives have a positive effect on the intestine; for example, they promoted villi and epithelial cells growth and development in carp, Atlantic salmon (*Salmo salar L*), and white shrimp [17,40,41]. In the present study, the intestine exhibited an orderly and tight epithelial layer, connective tissue layer, and muscle layer without edema after the crayfish were fed with *S. cerevisiae*. This might be because it increased the nucleotide contents of intestinal cells, which promoted DNA and RNA synthesis in the intestinal mucosa and improved bowel morphology [42].

### 4.3. Immune Related Gene Expressions

Crayfish lack typical adaptive immunity and instead rely on their innate immune systems to combat pathogenic invasion [9]. Changes in the expression levels of immune-related genes are important indicators of the health state of crayfish, and the intestinal microbiota balance can promote the improvement of immunity [43]. In this work, toll-like receptors, prophenoloxidase, Crustin2, and lysozyme genes in hepatopancreases of crayfish were determined. Toll-like receptors and prophenoloxidase can transmit invasion signals and induce a series of downstream cascade reactions [10]. Crustin2 and lysozyme have important antibacterial and antiviral effects [11,12]. *S*. *cerevisiae* could enhance the activities of prophenoloxidase and toll-receptor expression in the hepatopancreas of giant freshwater prawn [44]. In addition, the marine yeast *Yarrowia lipolytica* could upregulate the expression of prophenoloxidase and lysozyme in white shrimp [45]. In this study, the crayfish fed with *S. cerevisiae* showed significantly increased expression of Lysozyme, prophenoloxidase, and toll-like receptor genes in the hepatopancreas. This might reflect the abundant *β*-1, 3-glucan in the cell wall of *S. cerevisiae*, which could promote the expression of immune genes in aquatic animals [46,47]. However, there was no significant effect on Crustin2 gene expression in the crayfish hepatopancreas of the *S. cerevisiae*-fed group. This might be related to different factors, such as the pathway of immune gene expression and the mechanism of Crustin2’s participation in immune regulation [48].

### 4.4. Intestinal Microbiota Analysis

Normally, the beneficial and harmful bacteria in the intestinal tract are in a dynamic balance [16]. This balance maintains the stability of the intestinal environment, which can help the body to inhibit the invasion of foreign pathogenic bacteria effectively and enhance the body’s non-specific immunity [49]. The dynamic balance of the intestinal microbiota of aquatic animals is affected by many factors, such as their own physiological state, feed composition, external stress, and water environment [50,51,52]. In this study, the distribution in PCoA suggested that the microbiota community structure in the intestines of crayfish after feeding with *S. cerevisiae* was more stable than that of the control group. From the perspective of phylum classification, the dominant phyla of both groups were *Firmicutes, Bacteroidetes, Fusobacteria,* and *Proteobacteria* in the intestines of crayfish. Compared with that in the control group, the abundance of *Firmicutes* increased, but that of *Bacteroidetes* and *Proteobacteria* decreased after feeding *S. cerevisiae* for 14 days and 28 days. As symbiotic bacteria in the intestines of crustacea, the prevalence of *Proteobacteria*, including harmful pathogens such as *Escherichia coli* and *Vibrio cholerae*, has been proposed as the potential signature of dysbiosis and risk of disease [53]. *Firmicutes* are defined as beneficial bacteria of the intestines, and their positive role in growth performance, immunity, digestion, and disease resistance of aquatic animals has been noted [54,55]. *S. cerevisiae* might improve intestinal health by increasing the proliferation of *Firmicutes* and inhibiting the growth of *Proteobacteria* [56].

At the genus level, the common bacteria in the intestines of crayfish are *Bacteroides*, *Cetobacterium*, and *Citrobacter* [3]. In the present study, *Bacteroides*, *Cetobacterium*, *Citrobacter*, and *Lactobacillus* were the dominant genera in the intestines of crayfish from both groups. *Cetobacterium* has been observed in high relative abundance in different freshwater fishes and could produce large quantities of vitamin B-12 [57]. *Lactobacillus* can reduce the intestinal pH, which might contribute to overcoming pathogen challenges [22]. *Bacteroides* could result in damage to intestinal tissues [58]. *Citrobacter* is an opportunistic bacterium in crayfish culture [6]. Although not statistically different, our results show that the relative abundances of *Cetobacterium* and *Lactobacillus* in the crayfish intestines from treatment group increased, and those of *Citrobacter* and *Bacteroides* decreased after feeding with *S. cerevisiae* for 14 days and 28 days. This might have been because the *S. cerevisiae* cell wall is rich in mannose oligosaccharides and *β*-glucans, which can promote the proliferation of probiotics [47]. In addition, the acidic substances produced after mannose oligosaccharide production are selectively fermented by intestinal microbiota, which will lower the pH value of the intestine and inhibit the growth of harmful bacteria [30].

### 4.5. Disease Resistance Analysis

*C. freundii* can invade shrimp and produce independent factors leading to disease outbreaks. [6]. A challenge test showed that *C. freundii* exhibited significant virulence to crayfish, with an LD50 value of 1.71 × 10^6^ CFU/mL [6]. In this study, after a 10-day challenge with a higher concentration (2.5 × 10^7^ CFU/mL) of *C. freundii*, the cumulative survival rate was 30–40% in the control group. Meanwhile, dietary supplementation of *S. cerevisiae* increased the survival rate in the challenge test to above 60%. It might be due to the increased abundance of intestinal probiotics and up-regulation of immune genes with defense and antimicrobial functions after feeding after *S. cerevisiae* feeding, thereby enhancing resistance to pathogens invasion and infection [59].

## 5. Conclusions

In this study, the addition of *S. cerevisiae* YFI-SC2 in diet at 10^7^ CFU/g positively influenced the growth performance, the immune response, the intestinal microbiota structure, and the resistance to pathogens in crayfish. As long-term fresh *S. cerevisiae* YFI-SC2 are fed in the diet, a more pronounced effect will be observed. Therefore, *S. cerevisiae* YFI-SC2 can be used as a potential probiotic in crayfish farming.

## Figures and Tables

**Table 1:** List of primers used in this study.

Genes	Primer Sequence (5′-3′)	GenBank Number
**For qPCR**		
18S-F	TGGTGCATGGCCGTTCTTA	MT829236.1
18S-R	AATTGCTGGAGATCCGTCGAC	
Crustin2-F	GGGAAGAAAAGCACAATGGT	GQ301202.1 [23]
Crustin2-R	GGTATGGAGGTCGAGACAGG	
Prophenoloxidase-F	AGGTGGATCAGCCAGCAGT	EF595973.1 [24]
Prophenoloxidase-R	CGTAGTCAGCAGCGGAGGT	
Lysozyme-F	GATTGCTTAGGGTGCTTGTGCGA	GQ301200.1 [23]
Lysozyme-R	GGGTTTGCCAGCTTCATTCCAGT	
Toll-like receptor-F	GACTTGTCCAAAAACGATATACG	KP259728.1 [25]
Toll-like receptor-R	TGCGTTACAGTAGTGAGCGAAT	
**For V3—V4 regions of 16S rRNA gene**		
338F	ACTCCTACGGGAGGCAGCA	
806R	GGACTACHVGGGTWTCTAAT	

**Table 2:** Growth performance of crawfish fed with different experimental diets for 14 and 28 days.

Parameters	Dietary Treatments			
	CG14d	TG14d	CG28d	TG28d
Initial weight (g)	13.72 ± 0.18	13.69 ± 0.21	13.72 ± 0.18	13.69 ± 0.21
Weight at sampling (g)	15.13 ± 0.32	15.45 ± 0.39	16.67 ± 0.38 a	17.43 ± 0.37 b
WGR (%)	10.13 ± 0.43	12.86 ± 0.57	21.61 ± 1.03 a	27.09 ± 1.87 b
SGR (%/d)	0.69 ± 0.04	0.81 ± 0.13	0.72 ± 0.05 a	0.86 ± 0.07 b
Survival rate (%)	95.83 ± 1.44	96.25 ± 1.25	92.08 ± 0.72	93.75 ± 1.25

Note: CG14d and CG28d represents the control group after 14 days and 28 days of feeding. TG14d and TG28d represents the treatment group (*S. cerevisiae* at 10^7^ CFU/g diet) after 14 days and 28 days of feeding. Each value represents mean ± SD (*n* = 3), and bars with different letters indicate a significant different by LSD test for each time point (*p* < 0.05).

**Table 3:** OTUs classification information and alpha diversity index of microbial community in the intestines of crayfish.

Sample ID	OTUs	Chao 1	Shannon
CG14d	170 ± 28 a	207.08 ± 15.89 a	2.33 ± 0.27 a
TG14d	241 ± 31 b	258.97 ± 28.48 b	2.97 ± 0.32 b
CG28d	171 ± 17 a	201.04 ± 9.75 a	2.42 ± 0.15 a
TG28d	228 ± 23 ab	250.16 ± 16.48 b	2.85 ± 0.39 ab

Note: each value represents mean ± SD (*n* = 3). Different letters are significantly (*p* < 0.05) different by the LSD test. CG14d, control group after 14 days of feeding; CG28d, control group after 28 days of feeding; TG14d, treatment group (*S. cerevisiae* at 10^7^ CFU/g diet) after 14 days of feeding; TG28d, treatment group (*S. cerevisiae* at 10^7^ CFU/g diet) after 28 days of feeding; OUT, operational taxonomic unit.

## Data Availability

The data presented in this study are available on request from the corresponding author.
